# Endovascular Treatment of a Ruptured Pseudoaneurysm of the Intercostal Patch after Descending Aortic Aneurysm Repair

**DOI:** 10.3400/avd.cr.20-00122

**Published:** 2020-12-25

**Authors:** Yoshikatsu Nomura, Ryota Kawasaki, Motoharu Kawashima, Hiroshi Tanaka, Hirohisa Murakami

**Affiliations:** 1 Department of Cardiovascular Surgery, Hyogo Brain and Heart Center at Himeji, Himeji, Hyogo, Japan; 2 Department of Radiology, Hyogo Brain and Heart Center at Himeji, Himeji, Hyogo, Japan

**Keywords:** pseudoaneurysm of intercostal patch, Marfan syndrome, TEVAR

## Abstract

Anastomotic pseudoaneurysm and patch aneurysm are life-threatening complications following thoracoabdominal and descending thoracic aortic aneurysm (DTAA) repair. The aortic wall tissue is fragile in patients with Marfan syndrome, who are at high risk of anastomotic pseudoaneurysm and patch aneurysms. We experienced a rare case of ruptured pseudoaneurysm of the intercostal patch after DTAA repair in a patient with Marfan syndrome. A hematoma was separated from the pseudoaneurysm caused by adhesion of the left lung after DTAA repair, which made diagnosis difficult. To prevent type II endoleak and achieve thoracic endovascular aortic repair, we treated the patent intercostal arteries by embolization.

## Introduction

Anatomic pseudoaneurysm and patch aneurysm are life-threatening complications following thoracoabdominal aortic aneurysm (TAA A) and descending thoracic aortic aneurysm (DTAA) repair. Various methods exist for reconstruction of the intercostal arteries during TAA A and DTAA repair. Several reconstruction techniques, including the island technique and graft inteposition, were reported.^1)^ The island technique allows reimplantation of as many intercostal arteries as possible at once.^1,2)^ However, this technique demonstrates an increased risk of aneurysmal change, especially among patients with connective tissue disorders, and this was seen in 22.7% of patients with Marfan syndrome.^3)^ We report our experience with endovascular treatment of a ruptured pseudoaneurysm of the intercostal patch after DTAA repair.

## Case Report

**Figure 1:**
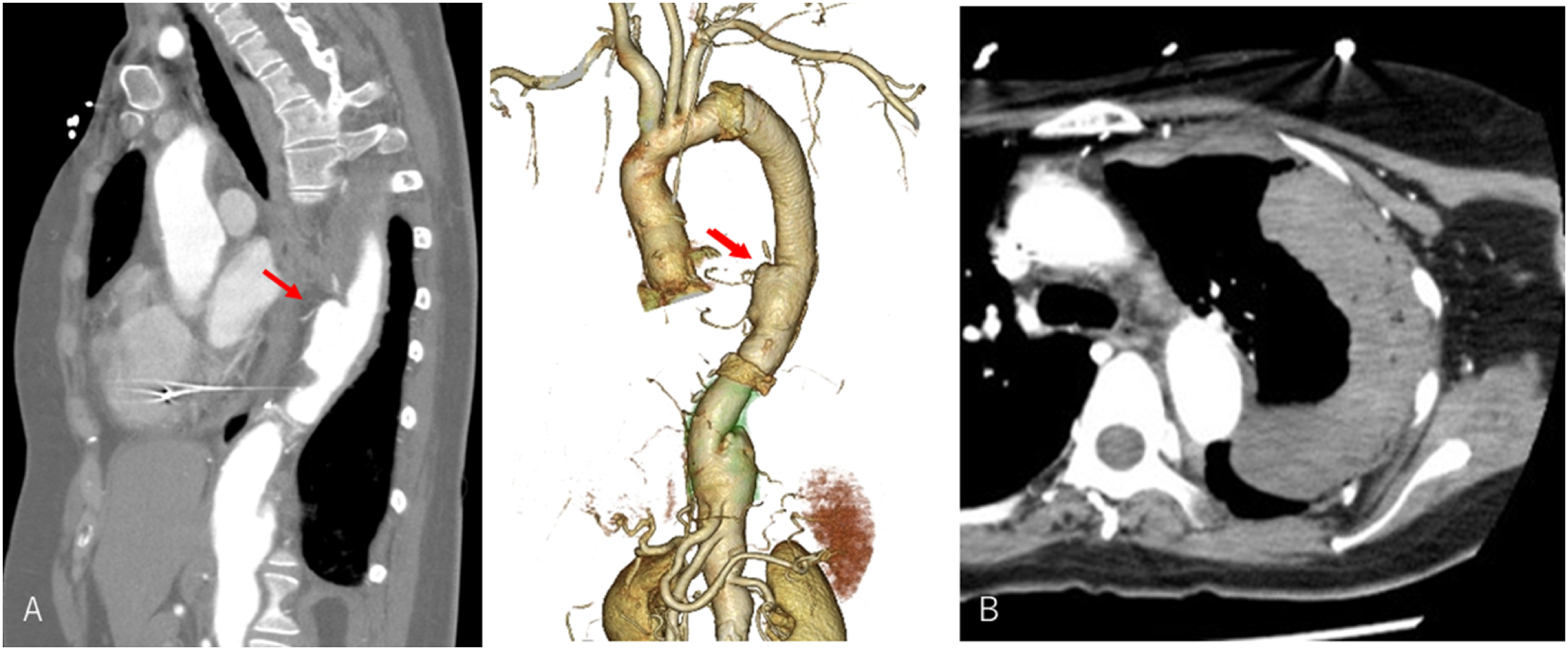
Fig. 1 (**A**) Contrast enhanced computed tomography image showing the intercostal patch aneurysm 32-mm in diameter (arrow). (**B**) The hematoma was separated from the aneurysm and accumulated in the apex of the lung.

**Figure 2:**
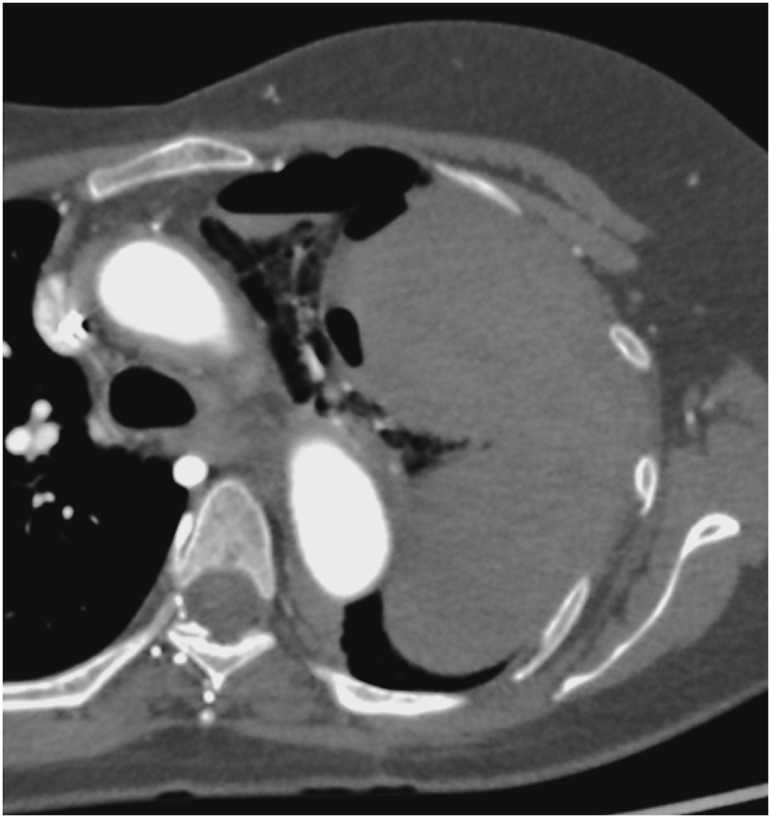
Fig. 2 The hematoma was seen gradually expanding.

**Figure 3:**
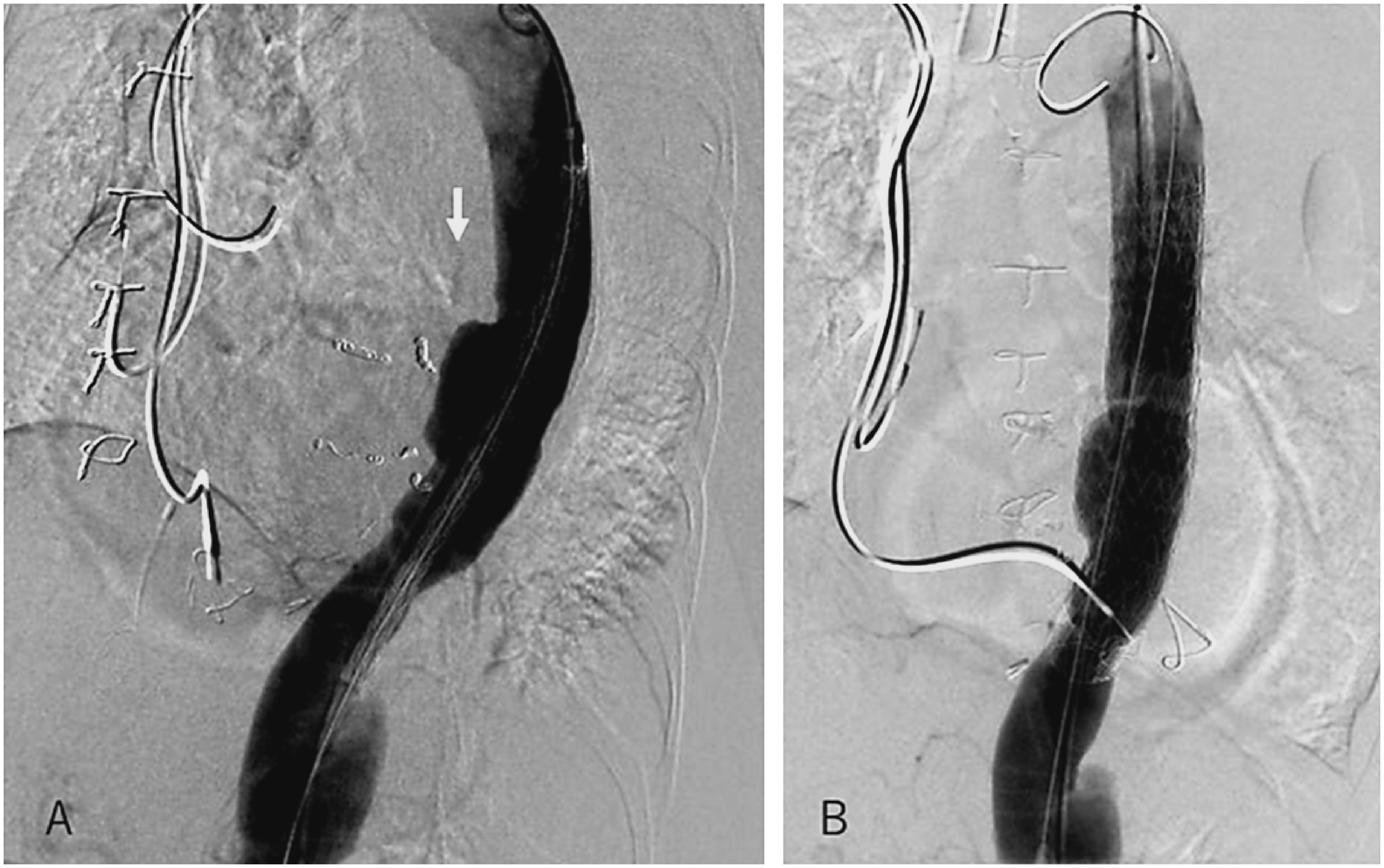
Fig. 3 (**A**) Angiography after embolization of the intercostal artery. A faint extravasation was observed at the head of the patch aneurysm (arrow). (**B**) No endoleak was recognized after embolization of the patent intercostal artery and endovascular stent graft of the ruptured pseudoaneurysm of the intercostal patch.

A 49-year-old woman with Marfan syndrome was admitted to our hospital complaining of hemoptysis. She presented with a history of valve-sparing aortic root reimplantation for annuloaortic ectasia and aortic valve regurgitation, descending aortic replacement with intercostal arteries patch reconstruction for Stanford type B aortic dissection, and permanent pacemaker implantation in another hospital. A chest computed tomography (CT) scan showed a hematoma in the apex of the left lung and a 32-mm intercostal patch aneurysm (**Fig. 1**). Two pairs of Th8 and 9 intercostal arteries were reconstructed in a patch, and all four arteries were patent. Because the patch aneurysm and hematoma were separated, we initially ruled out a rupture of the patch aneurysm. However, the hematoma gradually expanded, and the hemoptysis did not stop (**Fig. 2**). As a result, we reconsidered and diagnosed the case as a ruptured pseudoaneurysm of the intercostal patch aneurysm. Thoracic endovascular aortic repair (TEVAR) was the first-line treatment. However, due to retrograde flow from the intercostal arteries, the use of TEVAR alone was considered to carry a risk of a type II endoleak. Therefore, embolization of the patent intercostal arteries and subsequent TEVAR were planned. The patient was operated under general anesthesia. A motor evoked potential (MEP) monitor was also used to identify spinal cord injury. An 8 Fr sheath was inserted into the right femoral artery through the right inguinal incision, and angiography was performed at the level of the patch aneurysm. A very faint extravasation was observed at the head of the patch aneurysm. After selectively identifying the bilateral Th8 and 9 intercostal arteries using the micro catheter and micro guide wire, transarterial embolization (TAE) was performed using platinum coils. The MEP did not change during embolization, and to protect the spinal cord, the mean blood pressure was maintained above 80 mmHg. After TAE, the sheath was replaced with a 20 Fr DrySeal sheath® (W. L. Gore & Associates, Flagstaff, AZ, USA), and TEVAR was performed using the Conformable TAG® (TGU282815J, W. L. Gore & Associates, Flagstaff, AZ, USA) (**Fig. 3**). Hemoptysis disappeared immediately after TEVAR; however, bloody sputum persisted until postoperative day 4. The hematoma in the left thoracic cavity gradually disappeared, and the endoleak was not detected on postoperative CT findings. The patient was discharged on postoperative day 20. No adverse events, including infection, occurred up to 2 years postoperatively.

## Discussion

The reconstruction of the intercostal artery is an important surgical technique for protecting the spinal cord during TAA A and DTAA repair. Although various techniques were reported for reconstruction of the intercostal arteries, the island technique allows reimplantation of as many intercostal arteries as possible at once.^1,2)^ However, this technique carries an increased risk of aneurysmal change, especially among patients with connective tissue disorders. Kulik et al. reported that the incidence of the intercostal patch aneurysm was 7.1%.^3)^ In Marfan syndrome, the incidence rate was reported to be 22.7%.^3)^ Another study reported an incidence rate of 25% among patients with Marfan syndrome.^4)^ Hence, we avoid using the island patch technique in patients with Marfan syndrome.

Until a few years ago, open thoracic surgical repair was the only treatment for this aneurysm, and it required cardiopulmonary bypass and hypothermic circulatory arrest.^3)^ Although the result of conventional open thoracic surgical repair is good, endovascular treatment is minimally invasive, and it can be applied to cases where left thoracotomy is not possible. Several reports of endovascular treatment were published.^3–7)^ However, this treatment exhibits various demerits, such as development of a type II endoleak from the patent intercostal artery. In addition, landing the aorta with connective tissue disease is necessary. No report exists on the embolization of the patent intercostal arteries during TEVAR, but the occurrence of a postoperative type II endoleak due to the absence of embolization was observed.^3,7)^ In the present case, a postoperative type II endoleak was not observed by embolization of the patent intercostal artery prior to TEVAR. Treatment by endovascular repair using a physician-modified endograft with an intercostal artery directional branch was reported for intercostal patch aneurysm in a patient with Loyes–Dietz syndrome.^8)^ This technique is possible in cases with a large diameter of the intercostal artery and the presence of a landing zone. The rupture of pseudoaneurysm and patch aneurysm is rare but fatal condition. Other than the present case, a rupture has been reported in only one case.^7)^ In this patient, the hematoma was separated from the aneurysm owing to adhesion after left thoracotomy, which made diagnosis difficult. Due to the rupture of the pseudoaneurysm in this case, embolization of the intercostal artery was performed to prevent endoleak, which was time-consuming. If the type II endoleak remains, reaching the intercostal artery using the transcatheter approach is difficult after DTAA or TAA A repair. Therefore, if possible, the intercostal artery should be embolized.

## Conclusion

We reported a successful treated case of a ruptured pseudoaneurysm of the intercostal patch after TAA A repair. Although the occurrence of type II endoleak in the future is unclear, embolization of the intercostal artery prior to TEVAR is effective for endoleak prevention.

## Additional Note

This article was written based on enough informed consent and patient’s agreement.

The study was reviewed and approved by the institutional review bords (IRB number: 2-21).
